# What is New in the Management of Childhood Asthma?

**DOI:** 10.1007/s12098-018-2705-1

**Published:** 2018-06-09

**Authors:** Atul Gupta, Gayathri Bhat, Paolo Pianosi

**Affiliations:** 1 Department of Pediatric Respiratory Medicine, NHS Foundation Trust, King’s College Hospital, Denmark Hill, London, UK; 2 King’s College London, London, UK

**Keywords:** Asthma, Wheeze, Pediatrics/Children, Recent advance, FeNO, Adherence, Tiotropium, Omalizumab, Mepolizumab, Biological monoclonal antibody treatment, Temperature controlled laminar airflow device

## Abstract

Asthma still causes considerable morbidity and mortality globally and minimal improvement has been seen in key outcomes over the last decade despite increasing treatment costs. This review summarizes recent advances in the management of asthma in children and adolescents. It focuses on the need for personalized treatment plans based on heterogenous asthma pathophysiology, the use of the terminology ‘*asthma attack*’ over exacerbation to instill widespread understanding of severity, and the need for every attack to trigger a structured review and focused strategy. The authors discuss difficulties in diagnosing asthma, accuracy and use of Fractional exhaled nitric oxide both as second line test and as a method to monitor treatment adherence or guide the choice of pharmacotherapy. The authors discuss acute and long-term management of asthma. Asthma treatment goals are to minimize symptom burden, prevent attacks and (where possible) reduce risk and impact of progressive pathophysiology and adverse outcomes. The authors discuss pharmacological management; optimal use of short acting β_2_ agonists, long acting muscarinic antagonist (tiotropium), use of which is relatively new in pediatrics, allergen specific immunotherapy, biological monoclonal antibody treatment, azalide antibiotic azithromycin, and the use of vitamin D. They also discuss electronic monitoring and adherence devices, direct observation of therapy *via* mobile device, temperature controlled laminar airflow device, and the importance of considering when symptoms may actually result from dysfunctional breathing rather than asthma.

## Introduction

Asthma still causes considerable morbidity and mortality globally [1]. The recent Lancet commission [2] highlighted that our concept of the asthma is too simplified *i.e.*, that all asthma is considered the same and should be treated the same way. This belief has limited medical professionals to move forward and make significant progress in asthma management [2]. Recognition of the diversity of asthma and of the information that can be learned from outliers is leading us into management in the twenty-first century.

## Recognition of Asthma Phenotype: Need for Individualized Approach

Whereas asthma is often considered as one entity, the term should instead be used as a descriptive label for a collection of symptoms. It is important to understand the heterogeneity of the disease. Asthma is characterized by reversible airflow limitation, an oversensitive cough reflex and mucus hypersecretion. It may have no inflammatory component or there may be different patterns of inflammation, as there will be contributions from bacterial and viral infections that vary over time [2]. The Lancet commission suggested the importance of recognizing and defining the pathophysiology for an individual patient, *e.g*., eosinophilic airway inflammation and airway obstruction, with a focus on personalizing diagnosis and targeting care for treatable traits or pathology [2]. Fixed airflow limitation can be due to early life factors leading to structural changes or extramural causes [2]. Reversible limitation is due to an intramural cause: repeated contraction of airway smooth muscle, inflammatory mural edema or intraluminal airway secretions. Multiple forms of airway hyperresponsiveness exist and can even be present at birth with no evidence of allergy or inflammation. Airway inflammation in asthma is also heterogeneous. Eosinophilic inflammation has two known pathways; Early onset allergic eosinophilic airway inflammation (extrinsic asthma) and Late onset non-allergic eosinophilic airway inflammation (intrinsic asthma). Other pathways may exist and are yet to be discovered. Neutrophilic airway inflammation is another profile where at least two pathways have been found [2].

Phenotypic classifications vary globally and often cause confusion based on how the word phenotype has been defined. It is used to describe [3]:

- Any observable trait (morphological, biochemical, physiological, behavioral),
- Clinical grouping for wheeze/asthma (Tuscon grouping - early, late, persistent) (difficult/severe asthma)
- Specific disease entities (Atopic asthma, Viral-induced wheeze)

In this article authors have used the first definition of phenotype as any observable trait as it is the most encompassing. Box 1 shows classifications of asthma phenotypes [4].

**Box 1:** Classification of asthma phenotypes in children [4]

Symptom based	
• Age at onset	
• Natural history	
• Severity	
Trigger based	
• Allergic *vs*. non-allergic	
• Exercise induced	
• Viral triggered *vs*. multi triggered.	
Response to treatment	
• Corticosteroid responsive	
Inflammatory features (based on biopsy, induced sputum and bronchoalveolar lavage)	
• Eosinophilic	
• Neutrophilic	
Non-invasive markers	
• Exhaled nitric oxide	
• Exhaled breath condensate	
Pulmonary function tests	
• Fixed *vs*. bronchodilator-reversible airway obstruction	
• Bronchial responsiveness to exercise, cold air, chemical challenge.	

It is appreciated that invasive tests may not always be suitable for an asthma workup in children. However the more information that the clinician can obtain, the more targeted management can be. The recent Lancet commission [2] has advocated precision medicine where we should identify the pathophysiology and treatable aspects in an individual child or adolescent and personalize treatment rather than using a one size fits all approach to the treatment and secondary prevention of the asthma attacks.

## New in the Diagnosis of Asthma  – The Diagnostic Accuracy of Fractional Exhaled Nitric Oxide Testing

Diagnosing asthma in children is challenging and multiple tests including bronchodilator response and bronchial provocation challenge are used to help in the diagnosis of asthma in children with compatible symptoms, such as shortness of breath, wheezing, and cough. It is important to note that the asthma diagnosis remains clinical. No single diagnostic test exists.

Fractional exhaled nitric oxide (FeNO) concentration has been proposed to aid asthma diagnosis. A recent systematic review with meta-analyses evaluated the diagnostic accuracy of FeNO testing in children with suspected asthma [5]. They evaluated 43 observational studies addressing this question and concluded the FeNO concentration has moderate accuracy to diagnose asthma in individuals aged 5 y and older. The 2011 American Thoracic Society guidelines [6] recommended <20 ppb in children for low FeNO and > 35 ppb in children for high FeNO as the most useful cutoffs to make the determination that eosinophilic inflammation and response to corticosteroids would be unlikely or likely, respectively. Although different varieties of lower airway inflammation found in asthma have already been discussed, the fact remains that atopy is a significant risk factor in most childhood asthma [7]. Thus, FeNO measurement is likely to be particularly useful in diagnosis and management of asthma in children and adolescents. In summary, asthma can sometimes be difficult to diagnose, and FeNO can be helpful. The first diagnostic test in patients with suspected asthma should be spirometry with an assessment of bronchodilator response. If this test does not confirm the diagnosis, second-line tests (measurement of FeNO and bronchoprovocation studies) are recommended. Moreover, FeNO is sensitive to corticosteroid treatment and can therefore be used as a method to monitor treatment adherence or to guide choice(s) of pharmacotherapy.

## Dysfunctional Breathing

Before, and repeatedly during, treatment escalation for poorly controlled asthma, one must always re-evaluate presence of potential co-morbidities (Box 2) and should never forget that persistent symptoms may *not* be due to asthma. Frequently breathless patients have often learned a maladaptive breathing strategy causing, or perceived as, dyspnea – termed dysfunctional breathing [8]. A recent general review of the topic described some of these breathing strategies [9]. Dysfunctional breathing can co-exist with asthma [10, 11] and recognition of this condition is vital to (a) avoid iatrogenic, adverse effects from pharmacotherapy; and (b) open a path for non-pharmacologic intervention(s) [12, 13].

**Box 2:** Comorbidities of asthma

It is important to assess for the comorbidities as if underdiagnosed or undertreated, comorbid conditions can influence quality of life and asthma control.	
• Rhinitis	
• Rhinosinusitis	
• Nasal polyposis	
• Obesity	
• Obstructive sleep apnea	
• Gastro-esophageal reflux disease	
• Psychological stress, anxiety symptoms, depression	
• Dysfunctional breathing	
• Exercise induced laryngeal obstruction	

Perhaps the best known among the various types of dysfunctional breathing is exercise induced laryngeal obstruction (EILO) [14], formerly known as vocal cord dysfunction (among other synonyms). EILO is relatively easy to identify, but the clinician must first distinguish “wheeze” reported by patient or parent from stridor. Patients with EILO and asthma typically report difficulty with inhalation, so this feature in the history does not help differentiate these conditions, which becomes important since EILO can co-exist with asthma [10, 11]. Patients typically report a sensation of unsatisfied inhalation or “can’t get enough air” and may clutch their throat when saying so. Others may localize the site of “block” in the neck/throat if specifically asked. History is the key to identifying such patients, but the gold standard diagnostic test has become continuous laryngoscopy during exercise. Identification of other forms of dysfunctional breathing is not straightforward, and patients suspected of having it are best triaged and diagnosed *via* referral to a physiotherapist or speech-language therapist with requisite expertise. It is for this reason that obtaining as much objective information as possible, *i.e*., seeking evidence of reversible obstruction on spirometry, demonstrating airway hyperreactivity with bronchial provocation challenge, looking for evidence of lower airway inflammation (*e.g*., measurement of FeNO), and even chest imaging by CT scan showing mosaic attenuation, may be required. The true value of these tests lies in negative/normal results, which allows one to zero in on dysfunctional breathing as the culprit.

## New in the Management of Acute Asthma – Improving Treatment of Acute Asthma Attacks

An asthma attack should not be viewed as a temporary inconvenience but rather an event that may be associated with permanent damage to the lung [2]. It is important to understand the impact of terminology and asthma specialists recommend replacing the term exacerbation or flare up with attack as this will convey severity, future risk, and potential for death [2]. The presence of previous severe asthma attacks, psychological factors, and non-adherence to maintenance therapy are known factors contributing to “high risk” patients with asthma [15].

GINA (Global Institute of Asthma) guidelines [16] and BTS (British Thoracic Society)/SIGN (Scottish Intercollegiate Guidelines) guidelines [17] provide clear guidance on the management of acute asthma. Box 3 contains recent updates in GINA guidance. Briefly, there must be efficient assessment using objective measurements, appropriate use of delivery devices, not to hold back on oxygen use, and regular monitoring for response to treatment [18]. It is imperative that symptoms and signs are accurately assessed and differentiated between moderate, severe and life-threatening asthma attack. Treatment of a severe attack must include all of short acting β_2_-agonists, oxygen and steroids. Spacer devices have been shown to be equally if not more effective in mild to moderate attacks [19] and homemade spacers have been shown to be as good as commercial ones in some studies [20]. It is important to recognize that inhaled β2-agonist bronchodilators can occasionally exacerbate hypoxemia in patients with asthma, regardless of how they are administered. Hypoxemia can be lethal and pulse oximeters are advised particularly in primary care settings. These may be obtained cheaply and easily with probes appropriate for every age group [18].

**Box 3:** Recent updates in GINA guidance

• A continuous cycle of assessment, treatment, and review.	
• Asthma management to include self-monitoring of symptoms and peak flow, a written asthma action plan to recognize and respond to worsening asthma, and regular review of asthma control in partnership with a healthcare professional	
• Treatment with low dose ICS for most patients with asthma, even those with infrequent symptoms, to reduce the risk of serious exacerbations.	
• Sublingual or Subcutaneous immunotherapy to aeroallergens is not recommended for treatment of asthma in children.	
• When stepping down from low dose ICS, add on LTRA may help. There is insufficient evidence to step down to intermittent ICS with SABA.	
• When prescribing short-term OCS, remember to advise patients about common side-effects (sleep disturbance, increased appetite, reflux, mood changes)	
• Height should be checked at least yearly, as poorly-controlled asthma can affect growth and growth velocity may be lower in the first 1–2 y of ICS treatment	
• Effects of ICS on growth velocity are not progressive or cumulative.	
• Update of adherence strategies effective in real-life settings.	

*ICS* Inhaled corticosteroids; *LTRA* Leukotriene receptor antagonist; *OCS* Oral corticosteroids; *SABA* Short acting beta agonists

Research continues into additional bronchodilator therapy [inhaled magnesium sulphate (MgSO_4_) and intravenous MgSO_4_], and intravenous short-acting β_2_ agonists. Despite the overall lack of evidence, both forms of MgSO_4_ are widely used as part of the management of acute asthma in children. One meta-analysis involving three trials for intravenous MgSO_4_ in ED (Emergency department) indicated a number needed to treat (NNT) of 5 to prevent one hospital admission [21]. It is important to note the studies reviewed are difficult to compare directly with each other. Irazuzta et al. reviewed high dose MgSO_4_ continuous infusion *vs*. bolus MgSO_4_ in a prospective randomized ED study; this showed the high dose infusion group had lower lengths of stay and earlier discharges [21]. A Cochrane review in 2017 looked at 25 trials of varying quality which included both adults and children. Some individual studies suggest that those with more severe attacks of shorter duration may experience a greater benefit. Regardless, the Cochrane review concluded treatment with nebulized MgSO_4_ has not shown clinical benefit in recent well-designed trails [22]. Nebulised MgSO_4_ does not appear to be associated with an increase in serious adverse events [22].

One pilot study has shown the benefit of high-flow nasal cannula oxygen therapy compared to face mask oxygen therapy in the emergency department. They showed a reduction in respiratory distress measured *via* pulmonary score and hypoxemia, within the first 2 h of treatment and that it was safe and effective [23]. More research is required in this area.

Prednisolone is well used in the management of acute asthma, there has been research to assess the use of dexamethasone as an alternative based on its longer biologic half-life and improved palatability. It also appears to be better tolerated with reduced emesis. Research needs to continue to understand optimum dosing, duration and relative adverse effects [24].

Every asthma attack that a clinician encounters may be viewed as treatment failure and therefore should also trigger a structured review and focused strategy to reduce the risk of further attacks [18].

## New in Long Term Management of Asthma

Asthma treatment goals in children and adolescents are to minimize the short term effects (day-to-day symptoms, disturbed sleep, and activity limitation) and the risk of adverse asthma outcomes (attacks, persistent airflow limitation, and medication side-effects) [1]. It is imperative to optimize maintenance therapies, assess for the independent significant risk predictors at least once a year, like short acting β_2_-agonist overuse, not receiving inhaled corticosteroids (not prescribed, poor adherence, or poor inhaler technique), tobacco exposure, ongoing allergen exposure, psychosocial issues, comorbidities (Box 2) and poor lung function. Ensuring good quality, consistent parental education is vital.

## Pharmacological Management

### Should Inhaled Corticosteroids be Used Alongside Short Acting β_2_ Agonists in Initial Treatment for Asthma?

Asthma is an inflammatory condition and should be treated with anti-inflammatory agents (*i.e*., inhaled corticosteroids). Early treatment with inhaled corticosteroid is associated with better outcomes, reduced risk of asthma exacerbations and reduced risk of asthma related death. That said, it remains to be shown whether early (or later) intervention with anti-inflammatory treatment alters the natural history of the disease [25]. It is also important to recognize that regular short acting β_2_-agonist use leads to tachyphylaxis, rapid receptor tolerance, rebound bronchoconstriction, reduced response to short acting β_2_-agonist, as well as increased inflammation and responses to allergens [1].

### Long Acting Muscarinic Antagonist

Tiotropium is a long acting muscarinic antagonist (LAMA), which is well established for the treatment of chronic obstructive pulmonary disease (COPD). Acetylcholine, a chemical messenger is released from the cholinergic parasympathetic nerves in the lungs and causes airway smooth muscle contraction, proliferation of airway smooth muscles, mucus production, increase of ciliary beat frequency, and release of proinflammatory mediators by airway epithelial cells, proliferation of fibroblasts and vasodilation [26]. Tiotropium binds equally well to M_1_, M_2_, and M_3_ cholinergic receptors, but dissociates slowly from the M_1_ and M_3_ cholinergic receptors, hence the long duration of bronchodilator effect. It can be given once daily as the effect lasts for 35 h with maximum effect within 60 min [26, 27].

Tiotropium has shown promising results in five recent pediatric randomised controlled trials studies. Tiotropium delivered *via* the Respimat® Soft Mist™ inhaler has recently been approved for use as once-daily maintenance therapy for children with asthma over the age of 6 y in the USA (February 2017). GINA guidelines recommend tiotropium as an add-on therapy option at Steps 4 and 5 with a history of exacerbations, in patients aged 12 y and above. Findings of the large clinical trial program in children and adolescents across the spectrum of asthma severity have demonstrated that tiotropium Respimat® as add-on to inhaled corticosteroids, is a well-tolerated and efficacious bronchodilator, resulting in improved lung function [18, 27].

The safety of tiotropium delivered *via* Respimat® has recently been investigated in large clinical trials in adolescents and children with asthma [27, 28]. The efficacy of Tiotropium delivered *via* Respimat® is summarized in Table 1.

**Table 1:** Key efficacy findings from studies with tiotropium Respimat® in adolescents and children with asthma

Study program	Reference	Age	Asthma severity	Baseline therapy	Primary and key secondary endpoints	Key efficacy findings
RubaTinA-asthma® [29] (NCT01257230)	Hamelmann E, Bateman ED, Vogelberg C, et al. Tiotropium add-on therapy in adolescents with moderate asthma: a 1-year randomized controlled trial. J Allergy Clin Immunol. 2016;138:441–50. e8.	12–17 y	Symptomatic moderate	At least ICS	Peak FEV_1_ Trough FEV_1_	• Tiotropium add-on therapy improves lung function. • Improvement in peak FEV_1_ at wk 24 was statistically significant • 5mcg of tiotropium, adjusted mean difference 174 mL [95% confidence interval (CI) 76–272; *p* < 0.001]; 2.5 mcg of tiotropium, 134 mL (95% CI 34–234; *p* < 0.01). • Improvement was also noted in the trough FEV_1_ for tiotropium 5 mcg compared with placebo.
PensieTinA-asthma® [30] (NCT01277523)	Hamelmann E, Bernstein JA, Vandewalker M, et al. A randomised controlled trial of tiotropium in adolescents with severe symptomatic asthma. Eur Respir J. 2017;49:1601100.	12–17 y	Symptomatic severe	ICS + ≥1 controller	Peak FEV_1_ Trough FEV_1_ Mean FEF (Forced expiratory flow) (between 25 and 75% of forced vital capacity)	• Tiotropium 5mcg provided numerical improvements in peak FEV_1_ compared with placebo but was not statistically significant [90 mL (95% CI - 19 to 198; *p* = 0.104)]. • Statistically significant improvement in peak FEV_1(0–3 h)_ response with the 2.5 mcg dose [111 mL (95% CI 2–220; *p* = 0.046)] • The primary endpoint of the trial was not met as the efficacy of tiotropium 5mcg over placebo could not be demonstrated.
CanoTinA-asthma® [31] (NCT01634139)	Schmidt O, Hamelmann E, Vogelberg C, et al. Late-breaking abstract: once-daily tiotropium Respimat® add-on therapy improves lung function in children with moderate symptomatic asthma. Eur Respir J. 2016;48:PA4398.	6–11 y	Symptomatic moderate	At least ICS	Peak FEV_1_ Trough FEV_1_	• Significant improvement in peak FEV_1_ at wk 24 was observed with both doses of tiotropium (5 and 2.5 mcg), • Adjusted mean differences of 164 mL (95% CI 103–225) and 170 mL (95% CI 108–231) *vs*. placebo, respectively (*p* < 0.0001 for both comparisons). • Statistically significant improvements were also seen in trough FEV_1_ at wk 24 for both doses (*p* < 0.01).
VivaTinA-asthma® [32] (NCT01634152)	Szefler SJ, Murphy K, Harper T 3rd, et al. A phase III randomized controlled trial of tiotropium add-on therapy in children with severe symptomatic asthma. J Allergy Clin Immunol. 2017;140:1277–87.	6–11 y	Symptomatic severe	ICS + ≥1 controller	Peak FEV_1_ Trough FEV_1_ Mean FEF (Forced expiratory flow) (between 25 and 75% of forced vital capacity)	• Tiotropium 5mcg add-on therapy significantly improved peak FEV_1_ [139 mL (95% CI 75–203; *p* < 0.001)]. • A significant difference was observed in improvements in trough FEV_1_ response *vs*. placebo for the 5mcg dose.
NinoTinA-asthma® [33] (NCT01634113)	Vrijlandt EJLE, El Azzi G, Vandewalker M, et al. Safety and efficacy of tiotropium in children aged 1–5 years with persistent asthmatic symptoms: a randomised, double-blind, placebo-controlled trial. Lancet Respir Med. 2018;6:127–37.	1–5 y	Persistent asthmatic symptoms	At least ICS	Peak FEV_1_ Trough FEV_1_ Mean FEF (Forced expiratory flow) (between 25 and 75% of forced vital capacity)	• First study to assess the safety and efficacy of tiotropium in asthmatic children aged 1–5 y. • Tolerability of tiotropium was similar to that of placebo. • Interestingly, tiotropium showed the potential to reduce asthma exacerbation risk compared with placebo.

*ICS* Inhaled corticosteroids; *FEV*_*1*_ Forced expiratory volume in 1 s

In summary, clinical trials have shown improved lung function where tiotropium Respimat™ is used as add-on therapy to ICS (Inhaled corticosteroids) in patients with poorly controlled asthma. Tiotropium may be a useful and novel add-on treatment option, especially in patients where moderate-to-high ICS with or without a LABA (Long acting beta agonist) does not result in sufficient asthma control.

## Allergen-Specific Immunotherapy for Pediatric Asthma

Subcutaneous immunotherapy (SCIT) requires repeated injections with an allergen extract and is available for allergens such as grass and tree pollen and house dust mite. Sublingual immunotherapy (SLIT) is also available but is more effective as a high dose preparation than a low dose preparation. It has now become available with grass pollen allergen extract in a daily sublingual tablet [34]. Its steroid-sparing effect is an important benefit for patients who have to use these drugs in high doses and in long-term regimens. However, uncontrolled asthma remains a significant risk factor for side effects, and allergen specific immunotherapy should not be considered on safety grounds for patients who cannot achieve reasonably good control of symptoms with pharmacotherapy alone. Using GRADE (Grading of Recommendations Assessment, Development and Evaluation) criteria [34] van de Griendt et al. found limited benefit of SCIT and SLIT [23] and perhaps for this reason both GINA and BTS/SIGN asthma guidelines [16, 17] do not recommend allergen specific immunotherapy for treatment of asthma in children.

## Vitamin D for the Management of Asthma

There is considerable interest in the potential of administration of vitamin D to reduce exacerbation risk and improve asthma symptom control. The combination of antimicrobial, antiviral, and anti-inflammatory activity of vitamin D might decrease the risk of exacerbations, which are often precipitated by respiratory infection, is one theory to explain its efficacy. Evidence shows inadequate vitamin D status has been reported in children with asthma in a variety of settings [35]. Lower vitamin D levels in children are associated with worse asthma control, lung function and increased risk of exacerbations [36]. Gupta et al. demonstrated a negative relationship between airway smooth muscle mass and serum vitamin D levels in children with severe therapy resistant asthma [36]. Moreover, lower vitamin D levels were associated with lower lung function and increased asthma symptoms. Children with low serum vitamin D also had lower bronchoalveolar (BAL) levels of the anti-inflammatory mediator IL-10 [37]. When peripheral blood mononuclear cells (PBMC) from children with severe therapy-resistant asthma were stimulated with dexamethasone, release of IL-10 was significantly lower than that from control PBMC. However, addition of both dexamethasone and vitamin D3 resulted in a significant increase in IL-10 secretion [37]. These *in vitro* data suggest vitamin D enhances steroid sensitivity in severe therapy resistant asthma, and supplementation may not only help to improve steroid responsiveness, but also may impact airway smooth muscle remodeling. A recent meta-analysis in people with predominantly mild to moderate asthma suggests that vitamin D is likely to reduce both the risk of severe asthma exacerbation and health care utilization [38].

## Biological Monoclonal Antibody Treatment

Omalizumab, an anti-IgE, is the first monoclonal antibody therapy for severe asthma and is now licensed for moderate-to-severe allergic asthma in adults and children aged at least 6 y with IgE greater than 30 IU/L. Omalizumab has been shown to reduce exacerbations and hospital admissions in adults and children [39, 40]. The true mechanism of action for Omalizumab in children is not known but its response is predicted by elevated high type 2 biomarkers, and reduces virus-associated exacerbations [1, 40, 41].

Mepolizumab is licensed for neutralising antibodies that target interleukin-5. Mepolizumab has recently been approved for severe eosinophilic asthma in adults and, in some regions, adolescents. In adults, Mepolizumab has been shown to reduce severe asthma exacerbations [42] and reduce need for oral corticosteroid therapy [43]. It is important to note Mepolizumab is not licensed for children (<12 y) but is licensed for adolescents (aged 12–18 y) in some regions, though very few have been included in randomized controlled trials.

Within the next few years there might be 5 new classes of type 2 directed biologics available. However, a need exists to identify the most appropriate pediatric and adolescent severe asthma patients for these treatments and to better understand how to measure response [1].

## Macrolide Antibiotics

Macrolide antibiotics reduce exacerbation frequency in bronchiectasis by either their antibiotic or anti-inflammatory effects. Brusselle et al. [44] suggested benefits of macrolide antibiotics only in adults with non-eosinophilic inflammation. However, a recent study by Gibson et al. on 420 adults with persistent uncontrolled moderate-to-severe asthma, showed oral azithromycin decreased the frequency of moderate and severe asthma exacerbations [45]. Non-eosinophilic asthma has been described in children but is still poorly understood [1]. In children and young adults with asthma, the role of macrolide antibiotics is uncertain and use of long-term macrolides is not recommended at present.

## Temperature-Controlled Laminar Airflow (TLA) Device

The temperature-controlled laminar airflow (TLA) is a device which can be employed over a bed in a domestic environment and can result in massive reductions in allergen/particulate exposure. The device works by controlling nocturnal exposure to particulate exposure by delivering cooled and filtered air overhead of an individual with asthma during sleep. The greater density of the cooled air reverses the normal convection current, and displaces allergen-bearing particles out of the breathing zone [46]. Boyle et al. in a parallel group study for 12 mo in 282 subjects, aged between 7 and 70 y with house dust mite (HDM), cat or dog allergy showed significant improvement in asthma-specific quality of life (mini AQLQ and Pediatric AQLQ) and significantly decreased FeNO [47]. Importantly, there was a progressively greater significance of difference as the severity of asthma increased. In a *post hoc* analysis, patients with poorly controlled asthma [Asthma Control Test (ACT) score < 18] despite GINA stage 4 therapy had significant improvements in both symptom and sleep components of the AQLQ (Asthma Quality of Life Questionnaire) [46, 47].

## Electronic Monitoring and Adherence Devices

It is very well known that children do not want to and/or do not remember to take inhalers or medicines if they are feeling well, and so exacerbations on a background of poor adherence can be even more severe.

Guideline based asthma care is associated with good asthma control in majority of children when adhered to [48]. Adherence in asthma remains a major barrier to effective control [49, 50] as objective reviews (using electronic adherence monitoring) show majority of studies reporting mean adherence rates of 50% [50]. Multiple studies have shown that adherence rates of at least 75%–80% are required to significantly improve asthma control particularly in difficult to control asthma [48, 51, 52]. Reasons for non-adherence are intentional from children and families and non-intentional. They evolve from illness perceptions, medication beliefs, and practical adherence barriers [50].

With digital technology becoming prominent within healthcare, electronic monitoring devices appear to be a good novel approach to management. The device itself is a small electronic component which can be attached onto a metered dose inhaler or that is designed as part of the inhaler. Currently developed models can record that an actuation has occurred and the time, give an audio reminder for the child and parent, and transmit this data wirelessly, such that readings can be reviewed in real time and subsequently analyzed. It can be used in conjunction with a mobile device app which can measure other asthma triggers. Several randomized controlled trials have used an electronic monitoring device to assess adherence [53–57]. All showed a statistically significant increase in adherence for children that used the monitoring device.

Directly observed therapy and feedback *via* a mobile device is a new concept in inhaler technique monitoring [58]. Shields et al. noted no previous use of Mobile Direct Observation of Therapy (MDOT) in inhaler technique monitoring [58]. They conducted a pilot study using MDOT over a 6-wk period in 22 children with difficult to control asthma. Healthcare professionals evaluated inhaler technique using uploaded videos onto a secure platform and provided telephone instruction on improving inhaler use. Outcomes showed improvement in inhaler technique, asthma control and reduction in FeNO.

In summary, there is certainly a role for these technologies to assist monitoring, encourage adherence and differentiate poor response from poor adherence.

## Summary

There are important developments in the management of asthma that clinicians should be aware of and the authors have aimed to summarize them in this review. The authors have placed an emphasis on good clinical knowledge and primary prevention, understanding the heterogenic nature of asthma pathophysiology, and therefore the importance of personalized care and treatment plans. The outcomes of recent research on pharmacological treatments are beneficial when considering new or alternative treatment options particularly for severe or difficult to control asthma and authors have highlighted other alternative methods of management upon which there is much ongoing research.
